# Serologic Assessment of Public Health Action Needed for Trachoma, Coastal Ecuador, 2021–2024

**DOI:** 10.3201/eid3211.261024

**Published:** 2026-11

**Authors:** Everlyn Kamau, Lesly Simbaña, Stuart Torres, Nikolina Walas, Gretchen Cooley, Chabier Coleman, E. Brook Goodhew, Diana L. Martin, Hadley Burroughs, Manuel Calvopiña, William Cevallos, Sandra Vivero, Victoria Nipaz, Josefina Coloma, Gwenyth O. Lee, Gabriel Trueba, Joseph N.S. Eisenberg, Karen Levy, Benjamin F. Arnold

**Affiliations:** University of California, San Francisco, California, USA (E. Kamau, N. Walas, H. Burroughs, B.F. Arnold); Universidad de San Francisco de Quito, Quito, Ecuador (L. Simbaña, S. Torres, V. Nipaz, G. Trueba); Centers for Disease Control and Prevention, Atlanta, Georgia, USA (G. Cooley, C. Coleman, E.B. Goodhew, D.L. Martin); Universidad de las Americas, Quito (M. Calvopiña); Universidad Central del Ecuador, Quito (W. Cevallos, S. Vivero); University of California, Berkeley, California, USA (J. Coloma); Rutgers University, New Brunswick, New Jersey, USA (G.O. Lee); University of Michigan, Ann Arbor, Michigan, USA (J.N.S. Eisenberg); University of Washington, Seattle, Washington, USA (K. Levy)

**Keywords:** trachoma, *Chlamydia trachomatis*, serology, IgG, Pgp3 antigen, plasmid gene protein 3, elimination, bacteria, Ecuador

## Abstract

We evaluated the probability of needing public health actions against trachoma in Esmeraldas Province, Ecuador, by estimating seroconversion rates of *Chlamydia trachomatis* plasma gene protein 3 IgG in children. Seroconversion rates suggest high probability of the need for public health interventions in rural communities and motivate further assessment.

Trachoma is a blinding eye disease caused by infection with ocular strains of *Chlamydia trachomatis*; trachoma has been declared a public health concern by the World Health Organization and targeted for elimination by 2030 (*1*). Public health interventions for trachoma include surgery for trichiasis, mass administration of antimicrobial drugs, and programs to support facial cleanliness and environmental improvement (*2*).

IgGs against the plasma gene protein 3 (Pgp3) of *C. trachomatis* are sensitive markers of past infections, making them suitable for monitoring populations after mass administration of antimicrobial drugs (*3*). Pgp3 antibodies can be used to measure trachoma transmission intensity in population-based surveys (*4*). A global analysis of trachoma was used to develop methods that use the Pgp3 IgG seroconversion rate (SCR) to estimate the probability of needing public health actions (*5*).

The highest prevalence of trachoma is found in sub-Saharan Africa (*2*). Trachoma also remains a public health concern in remote areas of South America; elimination efforts by the Pan American Health Organization are ongoing (*6*). Little is known about trachoma in Ecuador. We recently identified high levels of incident seroconversion of Pgp3 antibodies within a birth cohort in Esmeraldas Province of coastal Ecuador (L. Simbaña et al., unpub. data, https://doi.org/10.64898/2026.02.16.26346420). Here, we describe augmenting those birth cohort samples by including samples from older children living in villages within the same region and estimated Pgp3 antibody SCRs. We compared those data with global SCR distributions to estimate the probability of needing public health actions against trachoma.

## The Study

We included samples from 2 studies. The ECoMiD longitudinal birth cohort study in Esmeraldas Province enrolled pregnant mothers during 2019–2022 along an urban–rural gradient that included Esmeraldas city (urban), Borbón (commercial center for the region), 4 communities accessible by road, and 4 communities accessible via the Santiago and Onzole Rivers (Appendix Figure) (*7*). Dried blood spots were collected during 2021–2024, when children were 6, 9, 12, 18, and 24 months of age (L. Simbaña et al., unpub. data). We also included serum samples collected from community-based, annual serologic surveillance of arbovirus transmission in Borbón; 5 other communities, 4 of which overlapped with the ECoMiD study areas (Maldonado, Colon Eloy, Timbiré, Santo Domingo); and Santa Maria, along the Cayapas River (*8*). Annual surveys were conducted during August–October and used age-stratified sampling, which oversampled 2–14-year-old children. 

We tested samples from 2–15-year-old children collected during the 2021 and 2022 surveys for Pgp3 IgG by using the Luminex platform (https://www.luminexcorp.com) at Universidad San Francisco de Quito (L. Simbaña et al., unpub. data). We combined data from both cohorts and stratified the communities into 2 groups, Borbón (799 samples) and rural villages (2,002 samples). We excluded birth cohort samples from Esmeraldas city (n = 355) because the city was not included in the arbovirus surveillance study. We summarized details of study setting, sample testing, and statistical analysis (Appendix).

We used SCRs to assess the probability of whether *C. trachomatis* transmission was at a level that warranted public health action (*5*). For the Borbón and rural village groups, we estimated SCRs by using a catalytic model and a constant force of infection (Appendix). We then estimated the probability (*P*) of populations falling into either action needed or action not needed categories (*5*) by using the equation *P*(Category|SCR) = *P*(SCR|Category) × *P*(Category). The action needed category identifies populations likely to experience disease sequelae and blindness from trachoma without intervention; the action not needed category identifies populations unlikely to have transmission intense enough to justify population-level interventions. We calculated the likelihood *P*(Category|SCR) as the empirical probability of SCR estimates from Ecuador relative to SCRs in a global trachoma serology dataset derived from well-characterized populations (*9*) (Appendix). We considered the serologic survey in coastal Ecuador to be the baseline and used an uninformative prior of category *P*(Category) = 0.5 (*5*). For each group, we also calculated the probability that the SCR was above or below specific thresholds to measure confidence levels. We used SCR thresholds of 2 and 4 per 100 person-years because those thresholds aligned with regions of high confidence (>90% probability) for action not needed (SCR <2) versus action needed (SCR >4) (*5*).

We analyzed a total of 2,801 dried blood spots from 1,243 children 0–15 years of age for Pgp3 IgG. The sample numbers were larger for younger children because of greater sample contributions from the birth cohort; fewer samples were obtained from 3–5-year-old children (Figure 1, panel A). Pgp3 IgG seroprevalence rose earlier and to a higher level in rural villages than in Borbón; IgG levels in both groups converged in children 13 years of age (Figure 1, panel B). Overall, Pgp3 IgG seroprevalence was 7.0% (95% CI 5.3%–9.0%; 56/799) in Borbón and ranged from 0% (95% CI 0%–9.3%) in Colon de Onzole to 31% (95% CI 19.1%–44.8%; 17/55) in Zancudo (Figure 1, panel C). Among 401 children (790 samples) who were 1–5 years of age, the SCRs per 100 person-years were 4.6 (95% CI 3.5–6.2) overall, 1.9 (95% CI 0.7–5.5) in Borbón, and 5.7 (95% CI 4.2–7.7) in rural villages per 100 person-years.

**Figure 1:**
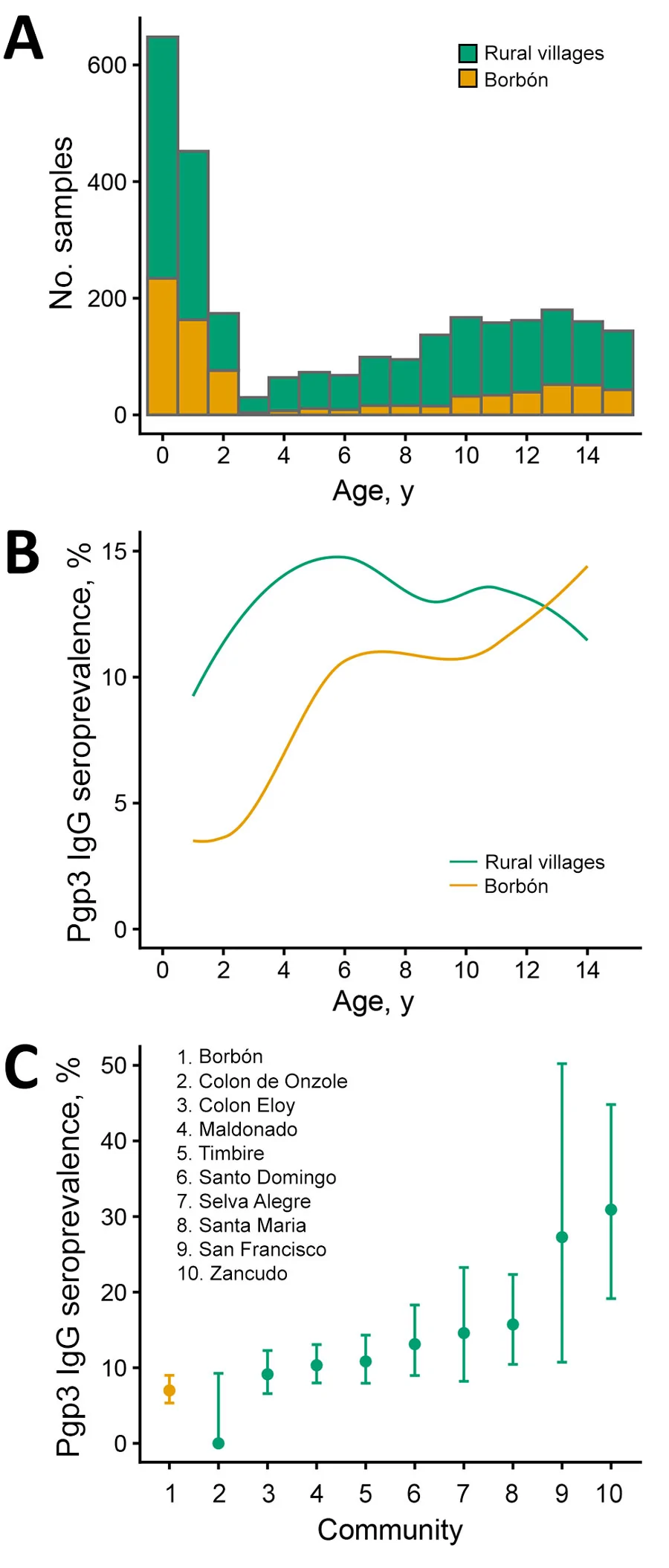
Seroprevalence of *Chlamydia trachomatis* Pgp3 IgG in rural areas and Borbón (commercial center) in Esmeraldas Province in study of serologic assessment of public health action needed for trachoma, coastal Ecuador, 2021–2024. A) Number of samples according to age and community type. A total of 2,801 samples were collected from 1,243 children who were 0–15 years of age; 790 samples were from 401 children 1–5 years of age. B) Seroprevalence according to age and community type estimated by using locally weighted regression, trimmed to 1–14 years of age to reduce edge effects. C) Seroprevalence among all children who were 0–15 years of age in Borbón and 9 rural villages. Error bars indicate 95% CIs. Pgp3, plasma gene protein 3.

When compared with SCR distributions from 34 global trachoma surveys, the probability that public health action was needed was 99.3% for the rural villages and 32.0% for Borbón (Figure 2, panel A). The probability that SCR was <2/100 person-years [i.e., *P*(SCR <2)], which represented high confidence for no public health action needed, was 0% for the rural villages and 52.4% for Borbón (Figure 2, panel B). Conversely, the probability that SCR was >4 [i.e., *P*(SCR >4)] was 91.3% for the rural villages and 8.8% for Borbón (Figure 2, panel B).

**Figure 2:**
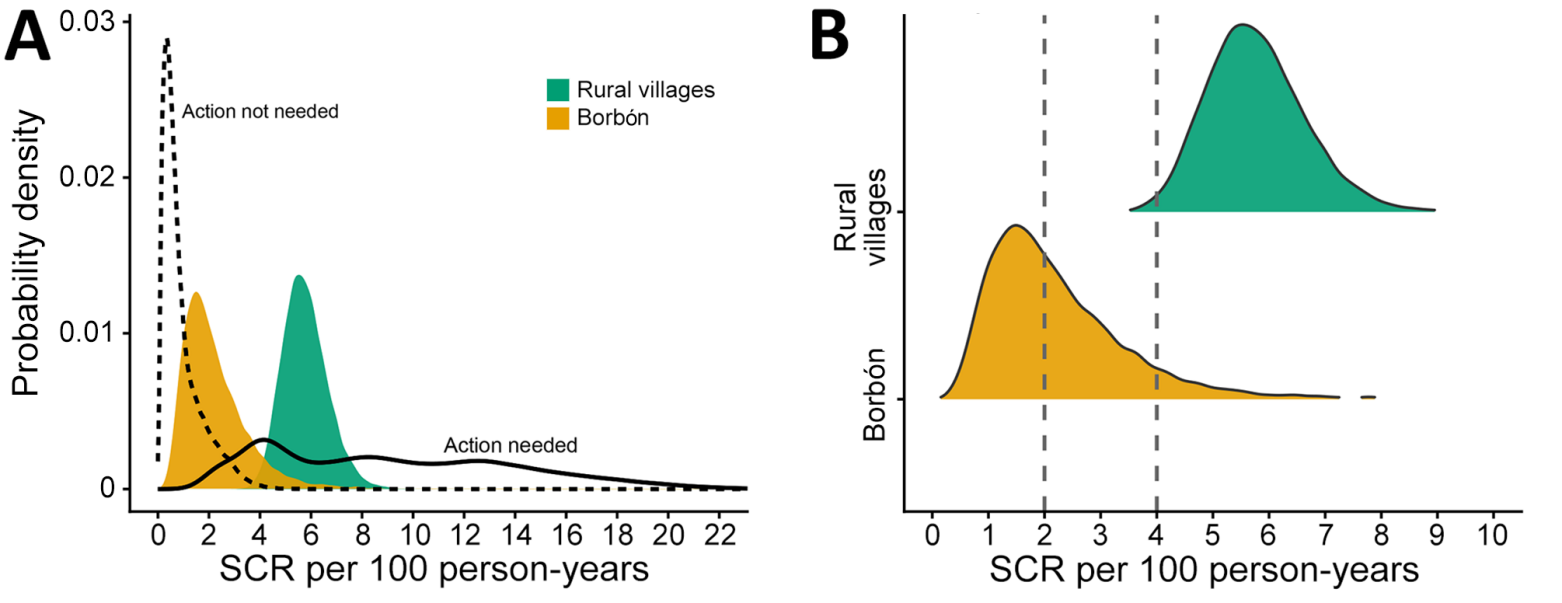
Model-based probability estimates of SCRs for IgGs against *Chlamydia trachomatis* plasma gene protein 3 in study of serologic assessment of public health action needed for trachoma, coastal Ecuador, 2021–2024. Estimates compared data from Esmeraldas Province in Ecuador and global trachoma serology distributions (*5*). A) Probability density of SCRs for Borbón (commercial center) and 9 rural villages estimated for children who were 1–5 years of age. We superimposed those data with pooled SCR distributions from populations classified as public health action not needed (dashed line) and public health action needed (solid line), according to public health responses for trachoma control in a global analysis across a range of disease endemicity (*5*). B) Ridgeline plot of SCR probability densities for Borbón and rural villages from panel A compared with illustrative thresholds of 2 and 4/100 person-years (vertical dashed lines). We identified SCR <2 as a region of >90% probability that public health action is not needed, whereas SCR >4 is a region of >90% probability that public health action is needed. Intermediate ranges of SCR that are >2 and <4 per 100 person-years could motivate additional testing (e.g., PCR testing for infection or consideration for further monitoring depending on programmatic context [*5*]). SCR, seroconversion rate.

## Conclusions

Pgp3 IgG SCRs in children from rural villages within the Santiago River basin of Ecuador suggest a high probability that public health action is needed for trachoma when compared with global SCR distributions (*9*). SCR estimates in rural villages fall within the range observed in districts with high levels of ongoing transmission that require interventions (*5*) and are consistent with estimates within disease-endemic districts of Ethiopia considered to have persistent, active trachoma (*10*).

Trachoma elimination efforts in the Americas include planned trachoma surveys in Ecuador; priority surveillance is within the Amazon basin (*11*). Our findings suggest that Esmeraldas Province should be considered as well. Although the World Health Organization has not yet set Pgp3 IgG SCR thresholds for stopping or starting interventions, we used SCRs of <2 and >4 as regions of high confidence to determine action not needed versus action needed. Additional monitoring of SCRs in Borbón is warranted, because the 95% CI of the SCR spanned the intermediate range of 2–4 cases/100 person-years.

The first limitation of our study is that we did not use a population-based probability sampling strategy that had many sampling clusters, as is commonly recommended for trachoma surveillance (*12*–*14*). Therefore, children who were 3–5 years of age might have been underrepresented, particularly in Borbón. Second, we did not characterize blood samples according to clinical signs of trachoma or ocular *C. trachomatis* infection. A natural next step would be to conduct standardized baseline surveys (*12*–*14*) to ascertain the presence of active trachoma and provide reference points to measure trachoma elimination progress.

Our analysis provides a generalizable example of using existing serosurveys to inform public health decision-making to support trachoma elimination. Pgp3 IgG SCRs can be used to identify communities at risk for persistent or reemerging trachoma transmission in the Americas, particularly in rural communities with limited access to safe water, sanitation, and hygiene.

Appendix Additional information for serologic assessment of public health action needed for trachoma, coastal Ecuador, 2021–2024.

## References

[1] World Health Organization. Validation of elimination of trachoma as a public health problem. 2016 [cited 2026 Jun 3]. https://www.who.int/publications/i/item/who-htm-ntd-2016.8

[2] Solomon AW, Burton MJ, Gower EW, Harding-Esch EM, Oldenburg CE, Taylor HR, et al. Trachoma. Nat Rev Dis Primers. 2022;8:32. 10.1038/s41572-022-00359-5 35618795

[3] Martin DL, Bid R, Sandi F, Goodhew EB, Massae PA, Lasway A, et al. Serology for trachoma surveillance after cessation of mass drug administration. PLoS Negl Trop Dis. 2015;9:e0003555. 10.1371/journal.pntd.0003555 PMC4340913 25714363

[4] Tedijanto C, Solomon AW, Martin DL, Nash SD, Keenan JD, Lietman TM, et al. Monitoring transmission intensity of trachoma with serology. Nat Commun. 2023;14:3269. 10.1038/s41467-023-38940-5 37277341 PMC10241377

[5] Kamau E, Ante-Testard PA, Gwyn S, Blumberg S, Abdalla Z, Aiemjoy K, et al. Characterizing trachoma elimination using serology. Nat Commun. 2025;16:5545. 10.1038/s41467-025-60581-z 40593530 PMC12216694

[6] Pan American Health Organization. Initiative for the elimination of trachoma in the Americas [cited 2025 Dec 10]. https://www.paho.org/en/initiative-elimination-trachoma-americas

[7] Lee GO, Eisenberg JNS, Uruchima J, Vasco G, Smith SM, Van Engen A, et al. Gut microbiome, enteric infections and child growth across a rural–urban gradient: protocol for the ECoMiD prospective cohort study. BMJ Open. 2021;11:e046241. 10.1136/bmjopen-2020-046241 34686548 PMC8543627

[8] Andrade P, Sosa-Moreno A, Vivero S, Nipaz V, Lee GO, Cevallos W, et al. The impact of Zika emergence in remote communities in northwestern Ecuador. J Infect Dis. 2024;230:e1058–66. 10.1093/infdis/jiae384 39082780 PMC11566036

[9] Kamau E, Ante-Testard PA, Colado J, Nash SD, Martin DL, Arnold BF, et al. Data resource profile: global trachoma serology data repository. Int J Epidemiol. 2025;54:dyaf183. 10.1093/ije/dyaf183 41208168 PMC12597674

[10] Nash SD, Astale T, Nute AW, Bethea D, Chernet A, Sata E, et al. Population-based prevalence of *Chlamydia trachomatis* infection and antibodies in four districts with varying levels of trachoma endemicity in Amhara, Ethiopia. Am J Trop Med Hyg. 2021;104:207–15. 10.4269/ajtmh.20-0777 33200728 PMC7790060

[11] Pan American Health Organization. Ecuador: partnership to eliminate trachoma as a public health problem [cited 2025 Dec 10]. https://www.paho.org/sites/default/files/2024-03/2024-cde-paho-canada-trachoma-public-health-ecuador.pdf

[12] World Health Organization. Design parameters for population-based trachoma prevalence surveys. 2018 [cited 2026 Jun 3]. https://www.who.int/publications/i/item/who-htm-ntd-pct-2018.07

[13] Harding-Esch EM, Burgert-Brucker CR, Jimenez C, Bakhtiari A, Willis R, Bejiga MD, et al. Tropical data: approach and methodology as applied to trachoma prevalence surveys. Ophthalmic Epidemiol. 2023;30:544–60. 10.1080/09286586.2023.2249546 38085791 PMC10751062

[14] Kamau E, Gass K, Harding-Esch E, Díaz MIS, Ante-Testard PA, Kello A, et al. Design considerations for incorporating serological monitoring into trachoma prevalence surveys. Am J Epidemiol. 2026;•••:kwag245; Epub ahead of print. 10.1093/aje/kwag245 42817805

