# Supplementary material for: Serologic Assessment of Public Health Action Needed for Trachoma, Coastal Ecuador, 2021–2024
Source: Emerg Infect Dis. 2026 Nov;32(11):1870–3. doi: 10.3201/eid3211.261024 (PMC13715890; doi:10.3201/eid3211.261024)
Supplement: Supplementary file 1 [file 26-1024-Techapp-s1.pdf]

Article DOI: https://doi.org/10.3201/eid3211.261024
EID cannot ensure accessibility for supplementary materials supplied by authors. Readers who have difficulty accessing supplementary content should contact the authors for assistance.

Serologic Assessment of Public Health Action Needed for Trachoma, Coastal Ecuador, 2021–2024

Appendix

Supplementary Methods

Study Setting, Sample Testing, and Statistical Analysis

ECoMiD was a longitudinal birth cohort study in Esmeraldas Province on the northern coast of Ecuador, conducted to examine interactions between gut microbiome conditions and enteric infections and how environmental conditions affect the development of the gut microbiome (*1*). The study enrolled women who were ≥18 years of age and in their third trimester of pregnancy (37 weeks of gestation) and who were identified through prenatal care visits and outreach in local health clinics. The study excluded women with known, high-risk pregnancies, those who anticipated moving in the next 6 months, and those who planned to have a nonvaginal (caesarean) delivery. The study enrolled pregnant mothers from 2019 through 2022 along an urban–rural gradient that included Esmeraldas city (urban), Borbón (commercial center), 4 communities accessible by road, and 4 communities accessible via the Santiago and Onzole Rivers (Appendix Figure). Children in the birth cohort were followed for their first 2 years of life; biologic samples were collected every 3 months. The study region is predominantly lowland tropical rainforest and converted agricultural land that has undergone gradual development since road construction increased in the early 2000s, which brought increased connectivity between urban Esmeraldas city, the town of Borbón, and rural villages and has led to changes in infectious disease transmission dynamics documented through the EcoDess research consortium (*2*).

Field staff collected dried blood spot samples from children up to 5 times at 6, 9, 12, 18, and 24 months of age. Dried blood spots were collected on filter paper, each with six 10 µL

extensions. The blood spots were then dried, individually sealed with desiccant in plastic bags, stored at –20°C in the field office before being shipped to the Universidad San Francisco de Quito at ambient temperature, and then stored at −20°C.

Samples from children enrolled after Ecuador's COVID-19 pandemic shutdowns were the focus of IgG testing, which spanned the 2021–2024 period. A single 10-µL equivalent from each participant's sample was eluted overnight at 4°C in Buffer B (1X phosphate-buffered saline, pH 7.2–7.4, 0.5% casein, 0,5% polyvinyl alcohol, 0.8% PVP, 0.3% Tween 20, 0.02% NaN_3, 3 µg/mL of *Escherichia. coli* extract). Eluates were further diluted with Buffer B to a final concentration of 1:400 for use in the multiplex bead assay.

Fifty microliters of the eluted samples were incubated for 1.5 hours with microsphere beads with coupled antigens in a custom 45-plex assay on the Luminex platform containing antigens to a broad set of enteric pathogens, vaccine preventable diseases, arboviruses, and neglected tropical diseases, including Pgp3 and Ct694 (*C. trachomatis*). Microsphere beads were diluted in assay buffer (1X phosphate-buffered saline, 0.5% bovine serum albumin, 0.05% Tween 20, 0.02% NaN_3) to provide 1,250 beads per well.

Samples were incubated for 45 minutes with a mixture of biotinylated mouse anti-human IgG (50 ng/well) and IgG4 (40 ng/well) from Southern Biotech (https://www.southernbiotech.com). This was followed by a 30-minute incubation with R-phycoerythrin–conjugated streptavidin (250 µg/well) from Invitrogen, Waltham, MA, USA, serving as a fluorescent marker. All incubations were performed at room temperature with agitation; triple washes using PBST buffer (phosphate-buffered saline with 0.05% Tween) were performed between each step. Finally, the beads were resuspended in 100 µL phosphate-buffered saline, and the plates were stored at 4°C until they were read the next day.

IgG bound to each antigen was measured by using median fluorescence intensity minus background (MFI-bg) on the Luminex platform with a MAGPIX instrument. Plates included Buffer B-only wells as blanks along with a negative control and 2 pooled positive controls at different concentrations for quality assurance. Final MFI-bg values were obtained by subtracting the Buffer B blank from each antigen's MFI values. Half (11 of 22) plates were run in duplicate to quantify within- and between-plate variability in MFI-bg values. Plates were repeated if

positive control monitoring targets fell outside 2 standard deviations of the mean in the Levey-Jennings control chart.

Seropositivity cutoffs for Pgp3 (MFI = 238) and Ct694 (MFI = 710) were determined by using external values from a receiver operator characteristic curve analysis of known positive and negative samples at the US Centers for Disease Control and Prevention. We focused primarily on Pgp3 responses to align with elimination surveillance studies that relied primarily on Pgp3 IgG and used Ct694 as an adjunctive measure (*3*,*4*).

We estimated seroprevalence as the proportion of positive samples with exact binomial (Clopper-Pearson) 95% CIs. We combined data from both cohorts and stratified the communities into 2 groups: Borbón (the commercial center for the region) and rural villages. We excluded birth cohort samples from Esmeraldas city (n = 355) because the city was not included in the arbovirus surveillance study. We estimated prevalence differences between the 2 groups (Borbón and rural villages) by using a linear-binomial model with robust standard errors clustered at the child level. All analyses were performed in R version 4.6.0.

Estimation of SCR within a Generalized Linear Model using Maximum Likelihood

The seroconversion rate (SCR) from a single-rate catalytic model, assuming no seroreversion, is closely tied to the slope of an age-seroprevalence curve. The SCR is equal to the slope of the age-seroprevalence curve divided by the complement of the seroprevalence at age A = a. It can be shown that the SCR based on this model can be estimated as the exponentiated intercept from a generalized linear model with binomial error structure and a complementary log-log link:

$$log[-\log(1 - P(Y = 1|A))] = log\lambda + logA$$

where Y represents individual level serostatus (1: seropositive, 0: seronegative or equivocal), A is the child's age in years, and λ is the SCR. The SCR provides an estimate of the force of infection, an epidemiologic parameter that denotes the rate at which susceptible persons in the population become infected. In this analysis, we did not allow for seroreversion in the SCR estimation.

Because some children contributed samples for repeated measurements, standard errors were estimated by using a Huber-White robust variance estimator clustered at the child level (*5*).

That is, we accounted for repeated measurements within children using child-level clustered robust standard errors.

Estimation of Probability of Public Health Action—Action Not Needed versus Action Needed

First, we downloaded posterior distributions of SCR estimates derived from a global trachoma serology dataset of well-characterized populations (Open Science Framework, https://osf.io/ykjc4; Dryad, https://datadryad.org/dataset/doi:10.5061/dryad.5qfttdzhx) (*6*). Those populations had been determined by a group of experts to either require further action (Action needed) or no further action (Action not needed) (*3*). Therefore, the downloaded data could be labeled according to those 2 categories.

Then, for the Ecuador data, we created a distribution of random values from the estimated log(SCR) and its standard error (SE):

$$\mathbb{R} = exp\big(rnorm(n, logSCR, SE[logSCR])\big) * 100$$

to create the likelihood that would be used in the calculation of probability as described below.

To calculate probability for each category of public health action, we used a mixture model framework applied to the distributions of SCR estimates. This approach assumes that an SCR estimate is drawn independently from a 2-component distribution of the 2 categories above, $k \in \{1, 2\}$. So, for each component or category (C_k: action not needed, action needed) and SCR estimate, $x \epsilon \mathbb{R}$, we computed the posterior probability, $p(C_k|x)$, by using Bayes' rule:

$$p(C_k|x) \propto p(C_k) * \frac{p(x|C_k)}{p(x)}$$

where $p(C_k)$ is the prior probability that a population is in category C_k. In this study, $p(C_k) = 0.5$ as we considered the Ecuador study to be a baseline survey. $p(x|C_k)$, is the likelihood evaluated as the empirical probability density function at each value x. In other words, the probability of x relative to the distribution of values comprising the category C_k.

$p(x)$ denotes the marginal likelihood or normalizing constant for the posterior density obtained by integrating the products of $p(x|C_k)$ and $p(C_k)$. That is, the sum of the products of the density function and prior probability for each k,

$$\sum_{k=1}^{2} \omega_k f_k(x|C_k)$$

or

$$p(x|C_{k=1})p(C_{k=1}) + p(x|C_{k=2})p(C_{k=2})$$

Estimating $P(SCR \leq c)$

Using the simulated likelihood $\mathbb{R}$ above, we calculated $P(SCR \leq c)$ as a cumulative distribution function or the empirical probability density $\mathbb{R} \leq c$.

References

1. Lee GO, Eisenberg JNS, Uruchima J, Vasco G, Smith SM, Van Engen A, et al. Gut microbiome, enteric infections and child growth across a rural–urban gradient: protocol for the ECoMiD prospective cohort study. BMJ Open. 2021;11:e046241. PubMed https://doi.org/10.1136/bmjopen-2020-046241

2. Eisenberg JNS, Cevallos W, Ponce K, Levy K, Bates SJ, Scott JC, et al. Environmental change and infectious disease: how new roads affect the transmission of diarrheal pathogens in rural Ecuador. Proc Natl Acad Sci USA. 2006;103:19460–5. PubMed https://doi.org/10.1073/pnas.0609431104

3. Kamau E, Ante-Testard PA, Gwyn S, Blumberg S, Abdalla Z, Aiemjoy K, et al. Characterizing trachoma elimination using serology. Nat Commun. 2025;16:5545. PubMed https://doi.org/10.1038/s41467-025-60581-z

4. Tedijanto C, Solomon AW, Martin DL, Nash SD, Keenan JD, Lietman TM, et al. Monitoring transmission intensity of trachoma with serology. Nat Commun. 2023;14:3269. PubMed https://doi.org/10.1038/s41467-023-38940-5

5. Zeileis A, Köll S, Graham N. Various versatile variances: an object-oriented implementation of clustered covariances in R. J Stat Softw. 2020;95:1–36. https://doi.org/10.18637/jss.v095.i01

6. Kamau E, Ante-Testard PA, Colado J, Nash SD, Martin DL, Arnold BF, et al. Data resource profile: global trachoma serology data repository. Int J Epidemiol. 2025;54:dyaf183. PubMed https://doi.org/10.1093/ije/dyaf183

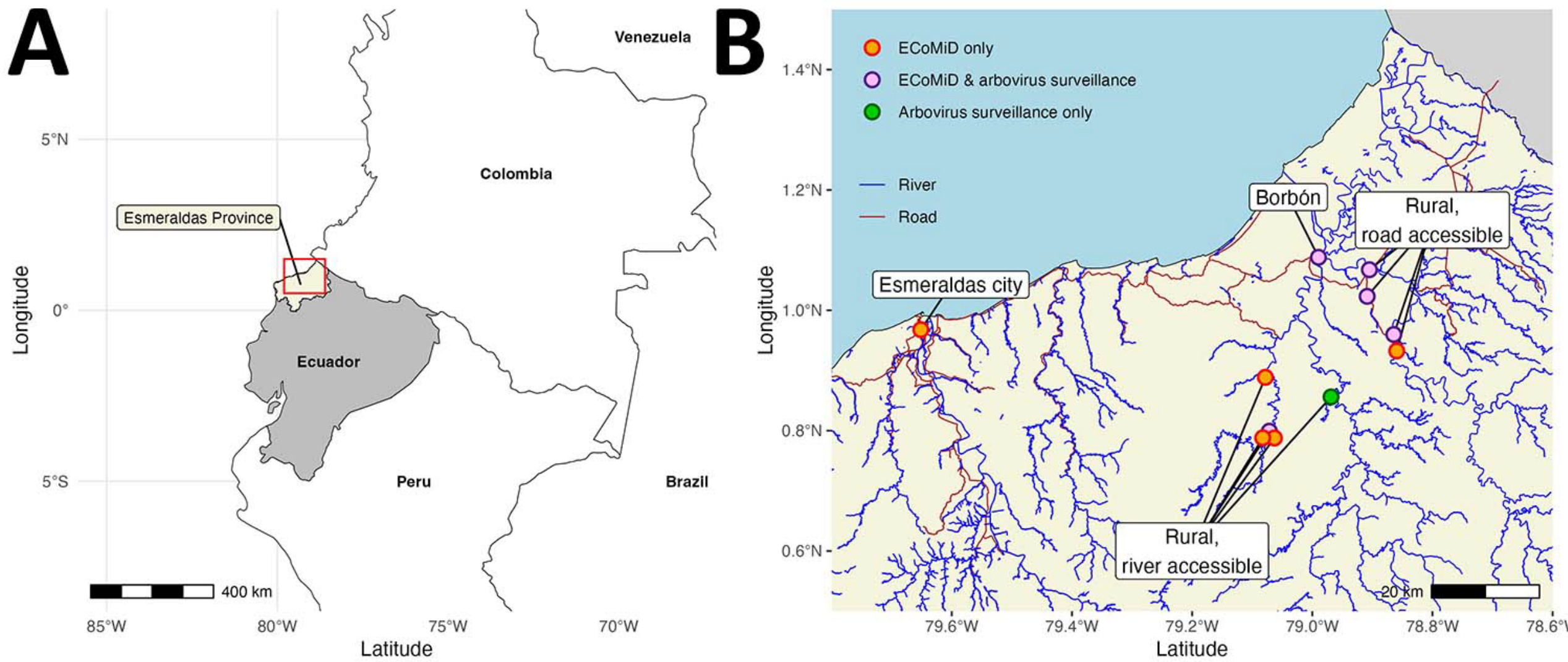

Appendix Figure. Study locations in Esmeraldas Province, Ecuador 2021–2024. A) Esmeraldas Province is in northern coastal Ecuador along the border with Colombia. B) Children were enrolled from Esmeraldas city (population ≈162,000), the town of Borbón (population ≈5,000), four road accessible rural villages (populations 500–1,000), and five river-accessible rural villages (populations 200–700). This study focuses on samples from Borbón and rural villages. Major rivers (blue) and major roads (brown) were provided by OpenStreetMap (https://www.openstreetmap.org).
